# A Robust Longitudinal Co-culture of Obligate Anaerobic Gut Microbiome With Human Intestinal Epithelium in an Anoxic-Oxic Interface-on-a-Chip

**DOI:** 10.3389/fbioe.2019.00013

**Published:** 2019-02-07

**Authors:** Woojung Shin, Alexander Wu, Miles W. Massidda, Charles Foster, Newin Thomas, Dong-Woo Lee, Hong Koh, Youngwon Ju, Joohoon Kim, Hyun Jung Kim

**Affiliations:** ^1^Department of Biomedical Engineering, The University of Texas at Austin Austin, TX, United States; ^2^Department of Biotechnology, College of Life Science and Technology, Yonsei University, Seoul, South Korea; ^3^Department of Pediatrics, Severance Fecal Microbiota Transplantation Center, Yonsei University College of Medicine, Seoul, South Korea; ^4^Department of Chemistry, Research Institute for Basic Sciences, Kyung Hee University, Seoul, South Korea; ^5^KHU-KIST Department of Converging Science and Technology, Kyung Hee University, Seoul, South Korea; ^6^Department of Medical Engineering, Yonsei University College of Medicine, Seoul, South Korea

**Keywords:** co-culture, gut microbiome, gut-on-a-chip, anoxic-oxic interface, host-microbiome interaction, microfluidics

## Abstract

The majority of human gut microbiome is comprised of obligate anaerobic bacteria that exert essential metabolic functions in the human colon. These anaerobic gut bacteria constantly crosstalk with the colonic epithelium in a mucosal anoxic-oxic interface (AOI). However, *in vitro* recreation of the metabolically mismatched colonic AOI has been technically challenging. Furthermore, stable co-culture of the obligate anaerobic commensal microbiome and epithelial cells in a mechanically dynamic condition is essential for demonstrating the host-gut microbiome crosstalk. Here, we developed an anoxic-oxic interface-on-a-chip (AOI Chip) by leveraging a modified human gut-on-a-chip to demonstrate a controlled oxygen gradient in the lumen-capillary transepithelial interface by flowing anoxic and oxic culture medium at various physiological milieus. Computational simulation and experimental results revealed that the presence of the epithelial cell layer and the flow-dependent conditioning in the lumen microchannel is necessary and sufficient to create the steady-state vertical oxygen gradient in the AOI Chip. We confirmed that the created AOI does not compromise the viability, barrier function, mucin production, and the expression and localization of tight junction proteins in the 3D intestinal epithelial layer. Two obligate anaerobic commensal gut microbiome, *Bifidobacterium adolescentis* and *Eubacterium hallii*, that exert metabolic cross-feeding *in vivo*, were independently co-cultured with epithelial cells in the AOI Chip for up to a week without compromising any cell viability. Our new protocol for creating an AOI in a microfluidic gut-on-a-chip may enable to demonstrate the key physiological interactions of obligate anaerobic gut microbiome with the host cells associated with intestinal metabolism, homeostasis, and immune regulation.

## Introduction

In the human colon, obligate anaerobic bacteria outnumber aerobic and facultative anaerobic bacteria by 2–3 orders of magnitude (Walsh et al., 2014). This commensal anaerobic microbiome constantly interacts with host cells and performs physiologically important functions including epithelial barrier function (Leslie et al., 2015; Chu, 2017), nutrient absorption (Krajmalnik-Brown et al., 2012), immune regulation (Luca et al., 2018), and the production of essential metabolic compounds such as short-chain fatty acids (SCFA) (Swanson, 2015). Interestingly, the host-gut microbiome ecosystem has been established in an anoxic-oxic interface (AOI) that is naturally created and maintained in the human colon. It has been known that the aerobic bacteria first colonize the luminal surface of the intestinal epithelium. Bacterial oxygen consumption is responsible for the creation of the oxygen gradient, eventually allowing the obligate anaerobes to colonize in the luminal microenvironment (Espey, 2013). The colonic microbiome has a complex spectrum from the oxygen-tolerant bacteria such as microaerophiles and obligate aerobes to the different types of anaerobic bacteria (i.e., facultative anaerobes, aerotolerant anaerobes, and obligate anaerobes; Albenberg et al., 2014). Since these colonic commensal bacteria substantially contribute to performing homeostatic and metabolic functions (Zhernakova et al., 2016), the establishment of the AOI with a defined transepithelial oxygen gradient is necessary to keep the microbial populations viable and functional.

It has been extremely challenging to recreate the human intestinal AOI *in vitro* and perform the host-gut microbiome co-culture because conventional aerobic *in vitro* cell culture models have failed to stably grow obligate anaerobic gut bacteria (Walker et al., 2014). For instance, a Transwell-based design was leveraged to co-culture the obligate anaerobic strain *Faecalibacterium prausnitzii* with intestinal epithelial Caco-2 cells, in which anaerobic and aerobic culture medium was applied to the apical and the basolateral compartment, respectively, to recreate a local AOI (Ulluwishewa et al., 2014). However, limited co-culture period (<8 h) and a lack of physiological flow and mechanical deformations substantially limited the longitudinal host-microbiome crosstalk (von Martels et al., 2017). A conical tube-based co-culture model was suggested to provide the AOI, where a solid agar compartment containing *F. prausnitzii* cells was incubated with a porous membrane lined by a monolayer of Caco-2 cells (Sadaghian Sadabad et al., 2015). However, in addition to the aforementioned restrictions of a static co-culture, the closed system in the design considerably hampered the necessary ventilation of the gases generated during the co-culture, which causes undesirable cracks in the agar.

Microfluidic models have also been proposed to recreate the AOI on-chip (Marzorati et al., 2014; Shah et al., 2016). However, these models separate the obligate or facultative anaerobic bacteria from the epithelial cell layer, preventing the direct host-microbiome interactions germane to the homeostatic functions of mucosal microbiome (Bäckhed et al., 2012). Furthermore, the lack of peristalsis-like mechanical movement, dynamic luminal flow, and fully differentiated 3-dimensional (3D) epithelial microarchitecture hamper the physiological legitimacy in modeling the human intestine. We previously developed a microengineered human gut-on-a-chip that permits robust co-culture of the living gut microbiome such as *Lactobacillus rhamnosus* GG (Kim et al., 2012), VSL#3 (Kim et al., 2016a,b; Shin and Kim, 2018b), and pathogenic enteroinvasive (Kim et al., 2016b) or non-pathogenic *Escherichia coli* (Bein et al., 2018; Shin and Kim, 2018a,b) in the 3D intestinal epithelium. However, it has not been fully verified if the longitudinal co-culture with obligate anaerobic gut bacteria is feasible in the gut-on-a-chip microphysiological system.

In this study, we modified the original design of the gut-on-a-chip with an increased height of the lumen microchannel to demonstrate the co-culture of obligate anaerobic bacteria with the intestinal epithelium by creating an anoxic-oxic interface-on-a-chip (AOI Chip). To validate the functionality of our *in vitro* AOI Chip, we chose two commensal obligate anaerobic bacteria, *Bifidobacterium adolescentis* and *Eubacterium hallii*, that have been known to syntrophically cross-feed and produce SCFAs (Belenguer et al., 2006). We also computationally and experimentally simulated the oxygen gradient microenvironment in the AOI Chip. Thus, we verified the feasibility of the AOI Chip by demonstrating the technically challenging co-culture of the human gut epithelium with obligate anaerobic gut microbiome.

## Materials and Methods

### Computational Simulation

A finite element method was used in COMSOL Multiphysics 5.3 (COMSOL Inc.). Two studies, the laminar flow fluid dynamics and the transport of diluted species, were coupled to compute the profile of oxygen concentration inside the microchannels in the presence or the absence of intestinal epithelium. To model fluid dynamics, the Navier-Stokes equation assuming incompressible fluid was applied, and convection of the fluid was also included (Mattei et al., 2014). As a boundary condition, the interfaces between the cell microchannels and the silicone polydimethylsiloxane (PDMS) layer were set as no slip conditions. The linear flow rate (m/s) was derived from a volumetric unit (μL/h) by reflecting the unit cross-sectional area of the cell microchannel. To simulate the transport of oxygen molecules, Fick's 2nd law was applied (Mehta and Linderman, 2006; Buchwald, 2009). The oxygen consumption rate was calculated based on the known oxygen consumption rate per intestinal epithelial cell (mol/s·cell) (Lin et al., 2018). The total number of epithelial cells in the AOI Chip was experimentally determined. The flux of oxygen through a PDMS layer was calculated by reflecting the thickness of the upper and the lower PDMS layers (Giulitti et al., 2013; Mattei et al., 2014). A PDMS porous membrane was simplified to consider as a thin diffusion barrier (thickness, 20 μm) by applying the diffusion coefficient of dissolved oxygen in the PDMS (Cox and Dunn, 1986). The standard mesh size was applied, and all simulations were performed with the assumption that the system is at 37°C. Parameters used in the simulation are provided in Table 1.

**Table 1:** Parameters used in COMSOL computational simulation.

**Parameter**	**Description**	**Quantity**	**References**
*D*_medium_	The diffusion coefficient of oxygen in medium	3.0 × 10^−9^ m^2^/s	Buchwald, 2009
*D*_cell_	The diffusion coefficient of oxygen in intestinal epithelium	2.0 × 10^−9^ m^2^/s	Buchwald, 2009
*D*_PDMS_	Diffusion coefficient of oxygen in PDMS	5.0 × 10^−9^ m^2^/s	Cox and Dunn, 1986
Cell density^*^	Epithelial cell number per unit volume	2.77 × 10^14^ cells/m^3^	
*Q_*O*2_*_cell_	The oxygen consumption rate of intestinal epithelium	8.64 × 10^−3^ mol/m^3^·s	Lin et al., 2018
*N*${}_{O_{2}}^{ext}$, *top*	Oxygen flux from the top PDMS layer	1.22 × 10^−7^ mol/m^3^	Giulitti et al., 2013
*N*${}_{O_{2}}^{ext}$, *bottom*	Oxygen flux from the bottom PDMS layer	6.10 × 10^−7^ mol/m^3^	Giulitti et al., 2013

* *Cell density was experimentally determined in this study*.

### Device Design and Microfabrication

An AOI Chip was fabricated using the soft lithography method as previously described (Kim et al., 2012; Huh et al., 2013). Cured PDMS (15:1, w/w; a mix ratio of the polymer base:curing agent; Dow Corning) was used to prepare the upper and lower microchannel layers demolded from a 3D printed cast made of MicroFine Green MR (Proto Labs). A microporous PDMS membrane was fabricated with PDMS (15:1, w/w) using a silicon wafer that conveys an array of micro-pillars (10 μm in diameter, 20 μm in height, 25 μm in spacing). After the uncured PDMS was poured onto the silicon wafer, covered with a thin fluoropolymer-coated polyester film (3M Scotchpak Release Liners), pressed with a 3 kg weight, then cured in an 80°C dry oven for at least 12 h. The upper PDMS layer was bonded to the PDMS membrane via plasma treatment (Femto Science) and subsequently bonded to the lower layer after corona treatment (Electro-Technic) after alignment under a stereoscope (Leica). The device setup was incubated at 80°C for the permanent bonding for longer than 12 h. Finally, a bent connector (hub-free stainless-steel blunt needle, 18G; Kimble Chase) linked to silicone tubing (Tygon 3350, ID 1/32″, OD, 3/32″, Beaverton) was inserted into each microchannel to supply oxic or anoxic cell culture medium or apply vacuum suction. A fabricated AOI chip was sterilized with 70% ethanol (v/v) and completely dried in a 60°C dry oven upon use.

### Microfluidic Culture

After the surface activation by UV and ozone treatment (UVO, Jelight Company, Inc.) for 40 min, microchannels were coated with an extracellular matrix mix (collagen I, 30 μg/mL, Sigma; Matrigel, 300 μg/mL, Corning) at 37°C for 1 h. Human intestinal epithelial Caco-2BBE cells (Harvard Digestive Disease Center) resuspended in Dulbecco's Modified Eagle Medium (DMEM, Gibco) containing 20% (v/v) heat-inactivated fetal bovine serum (FBS, Gibco) and antibiotics (100 U/mL penicillin and 100 μg/mL streptomycin, Gibco) were seeded into the upper microchannel (final cell density, 1 × 10^7^ cells/mL) and incubated for the cell attachment at 37°C in a humidified CO_2_ incubator for 1 h. The attached cells were further cultured under constant flow (at 50 μL/h, 0.02 dyne/cm^2^; Braintree Scientific) and mechanical deformations (10% in cell strain, 0.15 Hz in frequency; Flexcell International Corporation). For the microbial co-culture, the culture medium was replaced with the antibiotic-free medium 24 h before the bacterial cell seeding (final cell density, 1.0 × 10^7^ CFU/mL; CFU, colony forming unit). To create the AOI on-chip, anoxic medium pre-incubated in a glove box overnight was infused into the upper microchannel for 24 h prior to the microbial seeding. After microbial cell attachment on the apical epithelial surface without perfusion for 1 h, microfluidic co-culture culture was resumed under peristalsis-like flow and motions.

### Microbial Culture

Two obligate anaerobic bacteria, *Bifidobacterium adolescentis* (DSM 20083) and *Eubacterium hallii* (DSM 17630), were cultivated in the autoclaved tryptic soy broth (Difco) supplemented with 20% (v/v) FBS in an anaerobic glove box conditioned with the anaerobic gas mixture (5% O_2_, 5% H_2_, and 90% N_2_) without shaking at 37°C for 16 h. Bacterial culture broth of each strain was centrifuged at 10,000 × *g* for 1 min; then the cell pellet was resuspended with anoxic and antibiotics-free cell culture medium (final cell density, 1 × 10^7^ CFU/mL) for the seeding into the AOI Chip.

### Assessment of Epithelial Barrier Function

Epithelial barrier function was quantified by measuring transepithelial electrical resistance (TEER) using Ag/AgCl electrodes (A-M Systems) connected to an Ohm meter (Fluke Corporation). TEER value was calculated based on the equation, TEER (Ω·cm^2^) = (Ω_t_−Ω_blank_) × *A*, where Ω_t_ is resistance at each time point, Ω_blank_ is a resistance of a cell-free blank chip, and *A* is a surface area (cm^2^) of the microchannel lined by Caco-2 cells.

### Real-Time Microfluorimetric Detection of Oxygen

For the detection of oxygen *in situ* in the AOI Chip, platinum dendrimer-encapsulated nanoparticles (Pt-DENs) were used as an alternative to a peroxidase to catalyze the 10-acetyl-3,7-dihydroxyphenoxazine (Amplex Red reagent) into the resorufin in the presence of molecular oxygen. The Pt-DENs synthesized using the dendrimer-templating method (Crooks et al., 2001; Kim and Kim, 2014; Cho et al., 2018) were provided as a gift from Dr. Joohoon Kim. To detect the dissolved oxygen in the microfluidic channel, the cell culture medium pre-conditioned in either oxic or anoxic condition was mixed with Pt-DENs (3 μM) and Amplex Red reagent (500 μM), then added into the microchannel lined by the intestinal villous epithelium in the AOI chip. To create an AOI, the anoxic and oxic media were infused into the upper and lower microchannels, respectively, at 50 μL/h for 1 h. The device was imaged in a confocal microscope (DMi8, Leica) at an excitation and emission wavelength of 563 and 590 nm, respectively. An XZ-vertical scanning was performed in real-time to quantify the fluorescent intensity of the upper and lower channels at three different locations (inlet, middle, and outlet) across the microchannel in the device (Supplementary Figure 2). The oxygen tension as a function of fluorescent intensity detected by the confocal microscopy was determined by a calibration curve (Supplementary Figure 3B). Briefly, the absolute anoxic medium was prepared in a culture medium containing sodium sulfide (final concentration, 6.5 mM) and the reaction mixture (3 μM Pt DENs and 500 μM Amplex Red reagent; final concentration), then used to estimate the 0% oxygen tension. The saturated oxic medium in the given room temperature and pressure (1 atm) was prepared by mixing a conventional culture medium with the aforementioned reaction mixture to evaluate the 100% oxygen tension. The absolute anoxic medium was also used to obtain an initial kinetic profile of the oxygen dissolution from air into the culture medium in a 96 well plate (Supplementary Figure 3A). Finally, these anoxic and oxic media were independently introduced into the upper and lower microchannels in the AOI Chip, then placed in a confocal microscope to obtain micrographs that visualize the XZ scanning at different locations in the AOI Chip. The calibration curve (Supplementary Figure 3B) was used to quantitatively estimate oxygen tension in the upper and lower channels (Figure 3).

### Image Analysis

Immunofluorescence staining of the Caco-2 cells grown in the device was performed to characterize the epithelium of the AOI chip. When Caco-2 epithelium was fully grown under standard oxic culture conditions for a week, the anoxic and oxic media were perfused in the upper and the lower microchannels, respectively. After the AOI cultures for 3 days, cells were fixed (paraformaldehyde, 4%, w/v; Electron Microscopy Science), permeabilized (Triton X-100, 0.3%, v/v; Spectrum Chemical), and blocked [bovine serum albumin (BSA), 2%, w/v; HyClone] by sequentially flowing each reagent at 20 μL/h at room temperature for 15, 30, and 60 min, respectively. The primary antibodies for visualizing ZO-1 (Invitrogen; 50 μg/mL final concentration) and Mucin 2 (Santa Cruz Biotechnology; 40 μg/mL final concentration) were dissolved in the 2% BSA solution, then applied into the microchannel at room temperature for 1.5 h or at 4°C overnight. The secondary antibodies of anti-mouse 488 (50 μg/mL; Abcam) and anti-rabbit 650 (50 μg/mL; Abcam) dissolved in 2% BSA were introduced into the microchannel at room temperature in the dark for 1 h. After washing the microchannels with phosphate buffered saline (PBS; Ca^2+^ and Mg^2+^ free; Gibco), imaging analysis was performed with the confocal microscope (DMi8, Leica).

To perform Live/Dead cell viability assay to the epithelium, a mixture of Calcein AM (4 μM) and ethidium homodimer (EH)-1 (8 μM; ThermoScientific) was used. After villous epithelium was cultured in the AOI Chip for 7 days, the assay solution was perfused into the upper microchannel at 30 μL/h, 37°C for 30 min, then washed with PBS (Ca^2+^- and Mg^2+^-free). Images were taken under the confocal microscope (DMi8, Leica). Bacterial microcolonies were visualized by introducing the mixture of live (SYTO 9, 6 μM) and dead reagent (propidium iodide, 30 μM; Thermo Scientific) pre-incubated in the anaerobic glove box for 12 h prior to use. After Live/Dead assay, fluorescent images were immediately taken within 10 min to avoid epithelial staining. All the fluorescence imaging was performed using the TCS SPE confocal microscopy (DMi8, Leica) equipped with solid state excitation laser sources of 405, 488, 532, and 635 nm and a PMT. A 25 × objective (NA 0.95, Leica) was used for the differential interference contrast (DIC) and fluorescence imaging. The acquisition of an image was performed in the LAS X software (Leica). Bacterial cell viability was quantified based on the integrative intensity of the live and dead signal, read by the ImageJ. The following equation was used to calculate the viability; Viability (%) = *Int*_L_/(*Int*_L_+*Int*_*D*_), where *Int*_L_ indicates the integrative intensity of live cells and *Int*_D_ denotes the intensity of dead cells. Epithelial cell viability was quantified based on the live and dead cell number which was counted in ImageJ using the multipoint tool. The viability percentage was then calculated using the following formula; Viability (%) = *Cell*_L_/(*Cell*_L_+*Cell*_*D*_), where *Cell*_L_ stands for the number of live cells counted and *Cell*_D_ indicates the number of dead cells counted.

### Statistical Analysis

Two-tailed unpaired *t*-test was performed for statistical analysis between the experimental groups. One-way ANOVA with multiple comparison test was used to compare the means among three experimental groups in Figures 5D and 6D. All the statistical analysis was performed using GraphPad Prism 8 (GraphPad Software Inc.). All the plots and error bars were represented as mean ± standard error of the mean (SEM). Differences between experimental groups were considered as statistically significant when *p* < 0.05.

## Results

### Parameters for Computational Simulation of the AOI On-Chip

**Figure 1:**
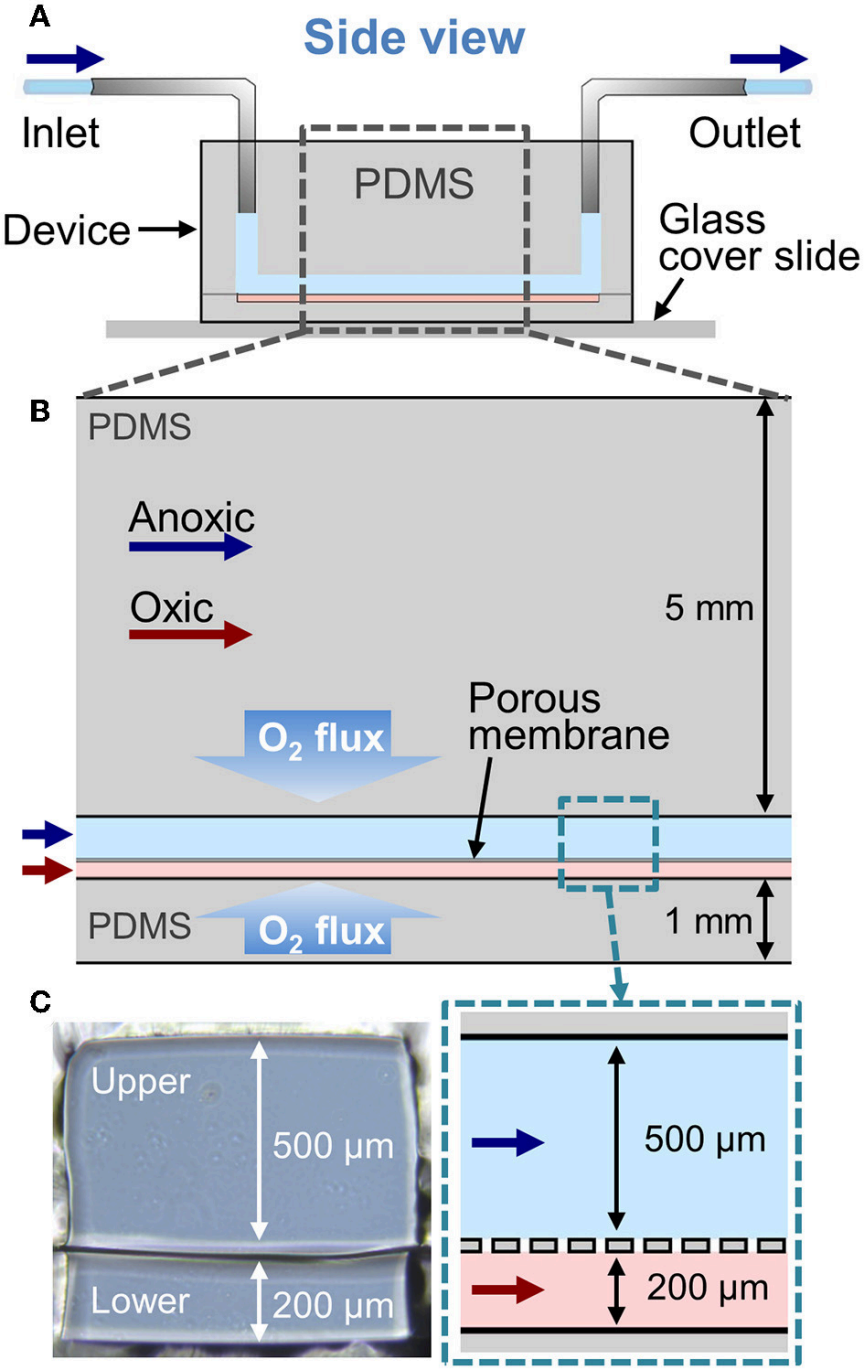
A configuration of the Anoxic-Oxic Interface-on-a-Chip. **(A)** A schematic of the side view of a microfluidic AOI Chip. The upper and the lower microchannel are indicated in light blue and pink, respectively. Arrows indicate the direction of culture medium in the microfluidic channels. **(B)** A zoom-in schematic of the AOI Chip shown in **(A)**. The upper and the lower cell microchannels are separated by a porous PDMS membrane (20 μm in thickness). Blue and red arrows represent the flow of anoxic and oxic culture media, respectively. **(C)** Cross-cut views of the microchannel area in the AOI Chip. A photographic (left) and a schematic (right) zoomed in from the dashed blue box in **(B)**.

To evaluate the feasibility of the AOI Chip model to create an oxygen gradient, we performed a simple 2D computational simulation to model the oxygen flux in the microfluidic device by varying experimental parameters such as the flow rate of culture medium, the diffusion of dissolved oxygen, and the presence or absence of an epithelial layer. Our microfluidic device made of PDMS has two microchannels separated by a thin PDMS porous membrane, in which the inlet and the outlet of each microchannel are connected to the silicone tubing (Figure 1A). The upper and the lower microchannels represent the lumen and the capillary side vasculature in the gut, respectively (Kim et al., 2016a). We constantly flowed the culture medium to both microchannels at the same flow rate (range, 50–200 μL/h, equivalent shear stress in the upper microchannel with 500 μm in height is 0.003–0.01 dyne/cm^2^). To create the AOI in the device, we perfused the anoxic cell culture medium to the upper microchannel (Figure 1B, light blue), whereas the oxic culture medium was flowed into the lower microchannel (Figure 1B, light pink). Since PDMS is gas permeable (Merkel et al., 2000), we applied the oxygen flux applied from the upper and the lower PDMS layers as 1.22 × 10^−7^ and 6.10 × 10^−7^ mol/m^3^, respectively. The different height of each microchannel (e.g., H_upper_ = 500 μm vs. H_lower_ = 200 μm) contributed to recreate controllable shear stress to the cells grown on the upper microchannel (Figure 1C). The geometry of the 2D microchannel, temperature (37°C), atmospheric pressure (1 atm), and the diffusion coefficient of dissolved oxygen in PDMS and medium were set constant in the simulation. The concentration of dissolved oxygen and the linear flow rate of culture medium in both microchannels were set as the key variables contributing to the formation of transepithelial oxygen gradient in the chip. Oxygen concentration applied in the upper and the lower microchannel was ranged from 0 (i.e., no oxygen) and 0.2 mol/m^3^ (i.e., oxygen saturated), respectively. The linear flow rate of culture medium was varied in a range of 27.8–111.2 μm/s (upper) and 69.5–278 μm/s (lower microchannel) as the cross-sectional area is different. Parameters used in the simulation are provided in Table 1.

### Theoretical Profile of Oxygen Gradient in the Cell-Free AOI Chip

**Figure 2:**
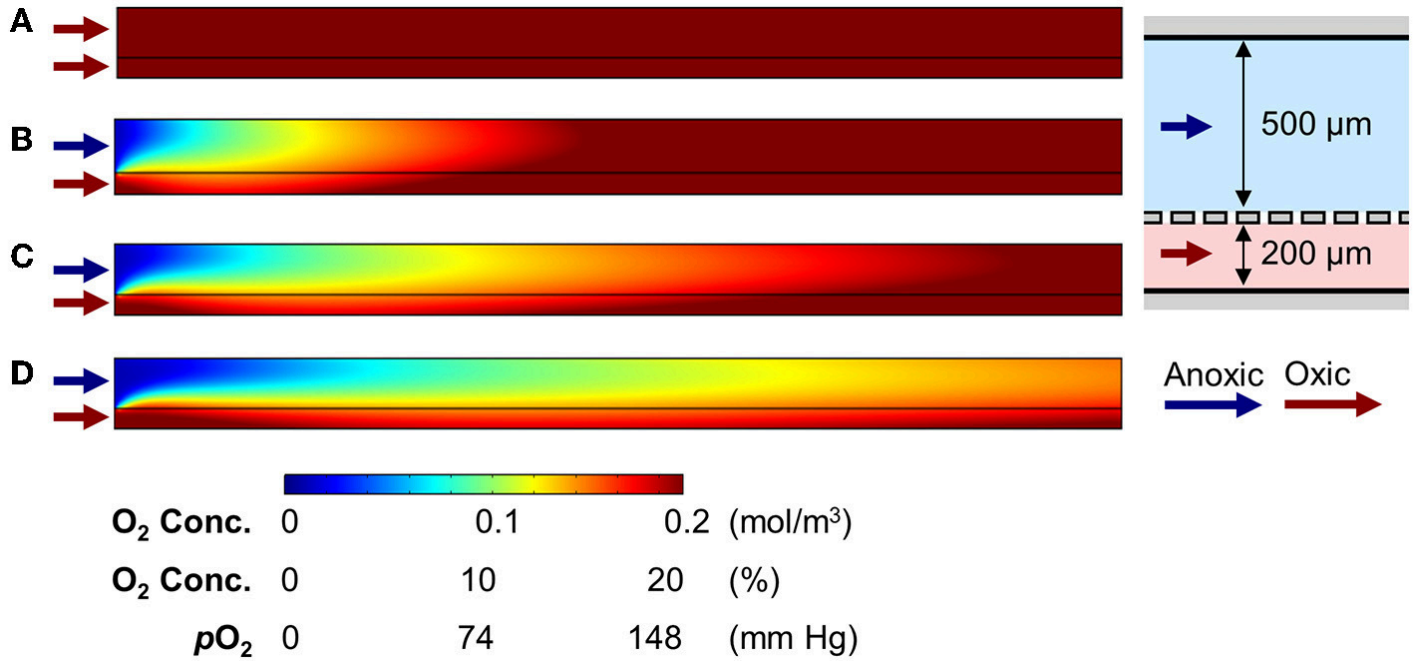
Generation of oxygen gradient in the cell-free AOI Chip by changing the flow rate of anoxic and oxic culture medium. **(A)** A heat map of the computational simulation displays the oxygen concentration gradient established in the microchannels of the AOI Chip when the oxic medium was flowed to both the upper and lower microchannels at 50 μL/h. **(B–D)** Heat maps of the oxygen gradient generated in the AOI Chip by flowing the anoxic (upper) and oxic medium (lower microchannel) at the flow rate of 50 μL/h (27.8 and 69.5 μm/s for upper and lower) **(B)**, 100 μL/h (55.6 and 138 μm/s for upper and lower) **(C)**, and 200 μL/h (111.2 and 278 μm/s for upper and lower) **(D)**. Blue and red arrows represent the flow of anoxic and oxic culture medium, respectively. A side-view schematic of the microchannels is placed in the right inset. A color bar at the bottom shows the scaled gradient of oxygen concentration with various convertible units.

First, we tested the effect of luminal and vascular flow rates on the recreation of AOI in the absence of an epithelial layer. The 2D simulation, in which we flowed the oxic medium through both upper and the lower microchannels at 50 μL/h (equivalent linear flow rate is 27.8 and 69.5 μm/s to the upper and the lower microchannels, respectively), demonstrates a steady influx of oxygen to both the upper and lower channels, and a steady-state aerobic condition is achieved in the AOI Chip (Figure 2A). When the anoxic and oxic medium was independently perfused to the upper and the lower microchannel at 50 μL/h, the AOI was partially created approximately 1/3 of the upstream of the microchannels (Figure 2B). We found that the oxygen supplied from the lower microchannel progressively diffused through the porous membrane toward the upper microchannel, so parabolic pattern of the convection of dissolved oxygen was observed predominantly in the upper microchannel. This parabolic oxygen gradient was further propagated from the upstream to the downstream of the upper microchannel when the flow rate was increased from 50 to 100 (Figure 2C) and 200 μL/h (Figure 2D) for both the upper and the lower microchannels (139, and 278 μm/s at the lower microchannel). However, in the absence of the colonic epithelial cell layer, the system failed to create the AOI in the given microenvironmental milieu.

### Profile of the Oxygen Gradient in the Epithelium-Containing AOI Chip

**Figure 3:**
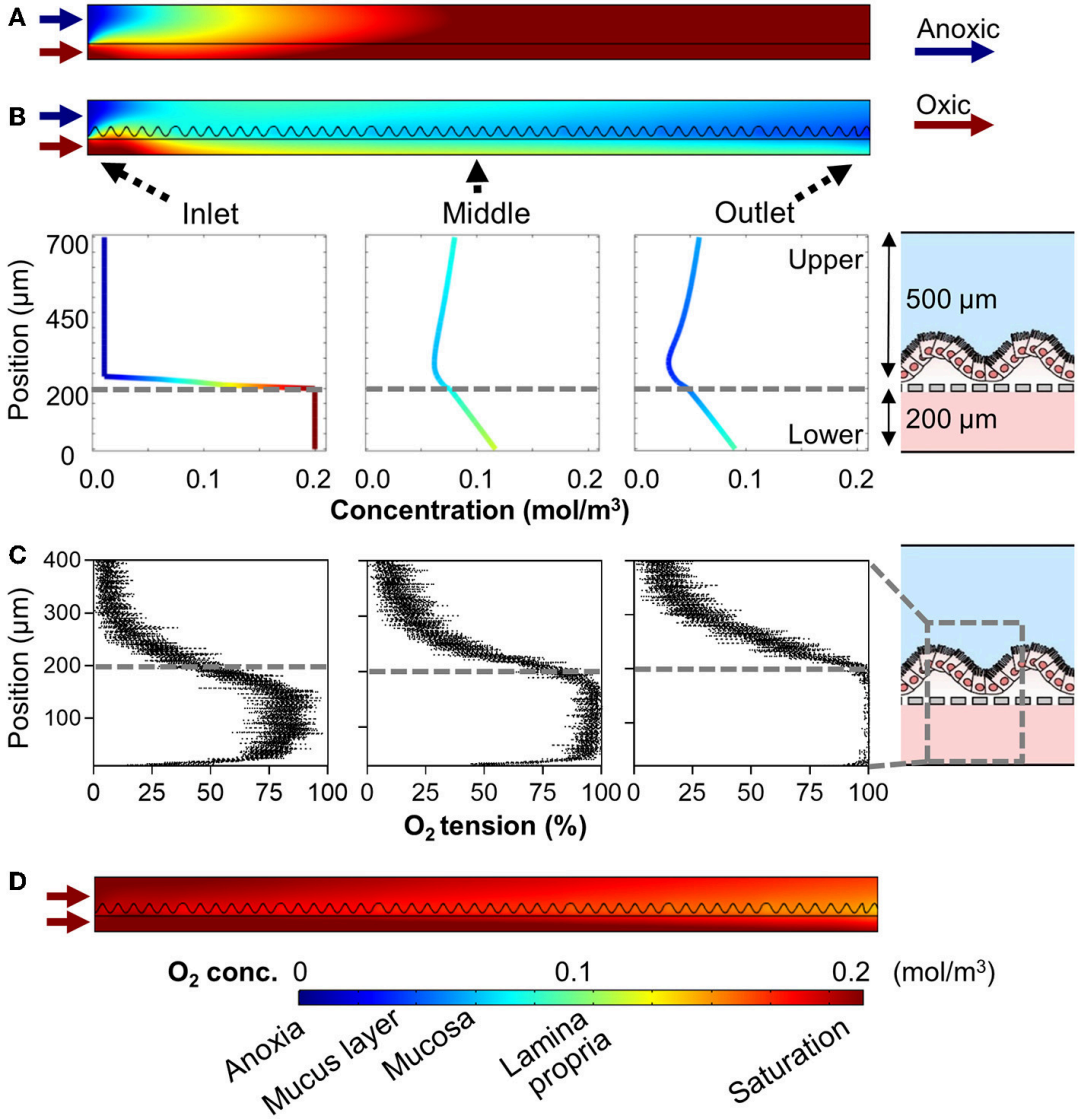
Profiles of the oxygen gradient generated in the AOI Chip in the presence of the 3D intestinal epithelial layer. **(A)** A heat map that computationally simulates the anoxic and oxic flow at 50 μL/h in the upper and the lower microchannels in the absence of an epithelial layer. This heat map is a replica of Figure 2B as a reference. **(B)** A heat map of the same computational simulation performed in **(A)** in the presence of a 3D intestinal epithelial layer (an undulating line in the upper microchannel). Colored line plots of the cell channel show the vertical concentration profile of the dissolved oxygen at three representative positions (Inlet, Middle, and Outlet). A relative position of the microchannel is set as 0 and 700 μm to the bottom of the lower microchannel and the top of the upper microchannel, respectively, in the y-axis. Gray dashed lines indicate the position where the porous PDMS membrane exists. A schematic in the right inset shows the XZ-configuration of the AOI Chip lined by the 3D epithelial layer. **(C)** Profiles of actual oxygen concentration generated at the inlet, middle, and outlet of the microchannels in the AOI Chip established at steady state. XZ line scan plots are obtained by the real-time microfluorimetric method via confocal microscopy after flowing the preconditioned culture medium at anoxic (upper) or oxic (lower microchannel) that contains Amplex Red reagent and Pt-DENs catalyst in the AOI Chip lined by the 3D epithelium for 1 h. The volumetric flow rate was fixed at 50 μL/h (*N* = 4). Gray dashed lines indicate the position of a porous PDMS membrane. A gray dashed box in the right schematic shows the location where the microfluorimetry XZ scanning was performed in the AOI Chip. **(D)** A heat map reveals the oxygen gradient in the presence of the intestinal epithelium where the oxic culture medium was flowed to both upper and lower microchannel at 50 μL/h. A color bar at the bottom shows the scaled gradient of oxygen concentration and the corresponding physiological conditions in the intestinal microenvironment. Blue and red arrows represent the flow of anoxic and oxic culture media, respectively.

Next, we performed an additional computational simulation to see the effect of 3D epithelial microarchitecture that constantly consumes the oxygen in the colonic microenvironment (Supplementary Figure 1). When we adopted the same boundary conditions applied to the cell-free simulations in Figure 2B (e.g., linear flow rates are 27.8 and 69.5 μm/s at the upper and lower microchannels, respectively) to the epithelium-containing AOI Chip, the oxygen profile in the upper microchannel was remarkably maintained the hypoxic condition in the mid-stream location of the microchannel, whereas the down-stream was nearly anoxic condition at steady-state (Figure 3B, upper). Individual line plots at the upper-, mid-, and down-stream regions showed that the anoxic gradient across the vertical crosscut of the microchannel was successfully recreated (Figure 3B, lower). In addition to the computational simulation, actual oxygen gradient generated in the epithelium-containing AOI Chip was visualized in real-time using a fluorescent oxygen indicating reagent (Amplex Red) in concert with the platinum (Pt) dendrimer-encapsulated nanoparticles (Pt-DENs) (Kim and Kim, 2014; Ju and Kim, 2015) in the microchannels (Figure 3C and Supplementary Figure 2). As a catalyst that converts the dissolved molecular oxygen to reactive oxygen species, we used the Pt-DENs that exhibit peroxidase-like activity comparable to a conventional horseradish peroxidase assay (Supplementary Figure 3). In the presence of reactive oxygen species, the Pt-DENs catalyze the oxidation of the Amplex Red reagent to produce fluorescent resorufin. By performing an XZ-vertical scanning of the AOI Chip via confocal microscopy, we quantitatively estimated the level of oxygen tension in the AOI Chip in a spatiotemporal manner (Figure 3C and Supplementary Figure 3). This result suggests that an AOI is stably formed across entire microchannel in the presence of 3D villous epithelium. However, the presence of an epithelial cell layer was not sufficient to consume the dissolved oxygen and generate the AOI on-chip when the oxic medium was perfused to both upper and lower microchannels (Figure 3D), suggesting that the infusion of anoxic culture medium into the lumen microchannel is a necessary approach to recreate the AOI on-chip.

### Epithelial Functionality in the AOI Chip

**Figure 4:**
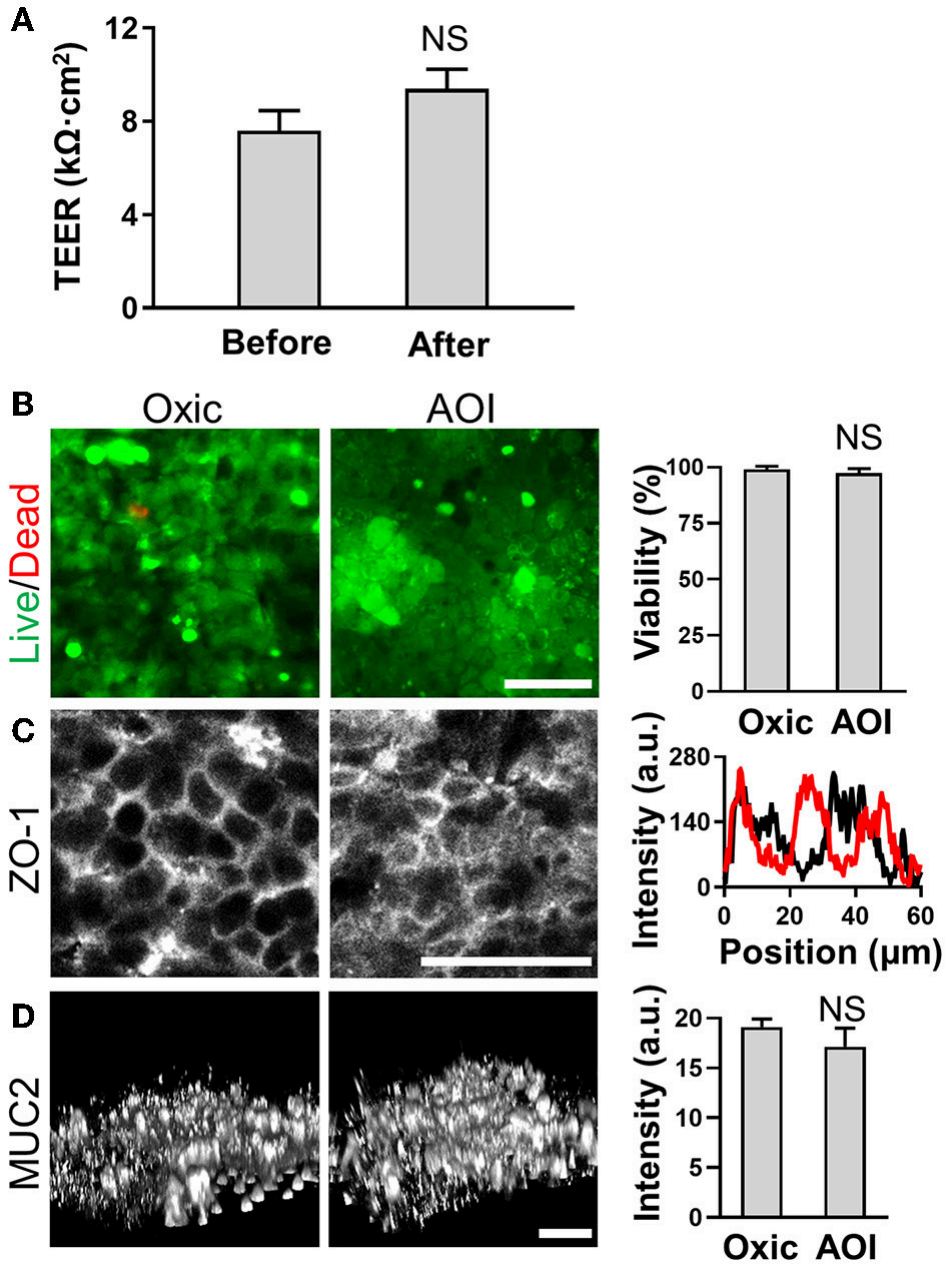
Epithelial barrier function and cell viability in the steady-state AOI on-chip. **(A)** The profile of the epithelial barrier integrity measured by TEER before (Before) and after (After; additional AOI culture for 24 h) the onset of AOI culture in the microfluidic device (*N* = 20). **(B)** The viability of the intestinal epithelium cultured in the presence (Oxic) or the absence (AOI) of oxygen in the upper microchannel whereas the lower microchannel is perfused with oxic medium for 7 days before the assays. The live (green) and dead cells (red) are visualized using Calcein AM (4 μM) and ethidium homodimer-1 (8 μM) in the overlaid confocal micrographs. Representative images are provided, and the quantification of cell viability is provided in the right chart (*N* = 6). **(C)** Expression and localization of the tight junction protein, ZO-1 (left) and the line scan at an arbitrary position of each immunofluorescence image (right). Black and red lines represent the oxic and the AOI, respectively. **(D)** Angled views of the 3D reconstruction of the expressed mucin 2 (MUC2) protein on the 3D intestinal epithelium (left) and the quantitative analysis (right; *N* = 3). NS, not significant. Bars, 50 μm.

Next, we verified the basic physiological functions of the intestinal epithelium in the AOI Chip by comparing to the epithelial cell functions grown in the oxic control. First, we conditioned the microfluidic device lined by the 3D villous epithelium by flowing the antibiotic-free anoxic and the oxic culture medium in the upper and the lower microchannel, respectively, for at least 12 h before seeding the microbial cells. It is noted that the device was incubated in a conventional humidified 5% CO_2_ incubator. Thus, the experimental setup is exposed to the atmospheric oxygen. We verified that the microengineered villi grown in the AOI Chip do not show any compromised epithelial barrier function assessed by measuring the transepithelial electrical resistance (TEER) before and after AOI conditioning for 48 h (Figure 4A). We also confirmed that the incubation of an epithelial layer in the AOI for 72 h also does not induce any cell death (Figure 4B), compromised expression of tight junction protein (ZO-1) (Figure 4C), or decreased level of mucin (MUC) 2-positive epithelium (Figure 4D) compared to the oxic control.

### Co-culture of Obligate Anaerobic Gut Microbiome in the AOI Chip

To demonstrate the stable co-culture of obligate anaerobic human gut microbiome with the human intestinal epithelial cells in the AOI Chip, we selected commensal *B. adolescentis* and *E. hallii* that have been known to synergistically produce SCFA in the human colon (Belenguer et al., 2006). More importantly, since these two strains are extremely sensitive to oxygen (Shimamura et al., 1992; Flint et al., 2007), it is substantially challenging to keep these microbial cells in the PDMS device lined by the gut epithelium that constantly requires oxygen (Zeitouni et al., 2015).

**Figure 5:**
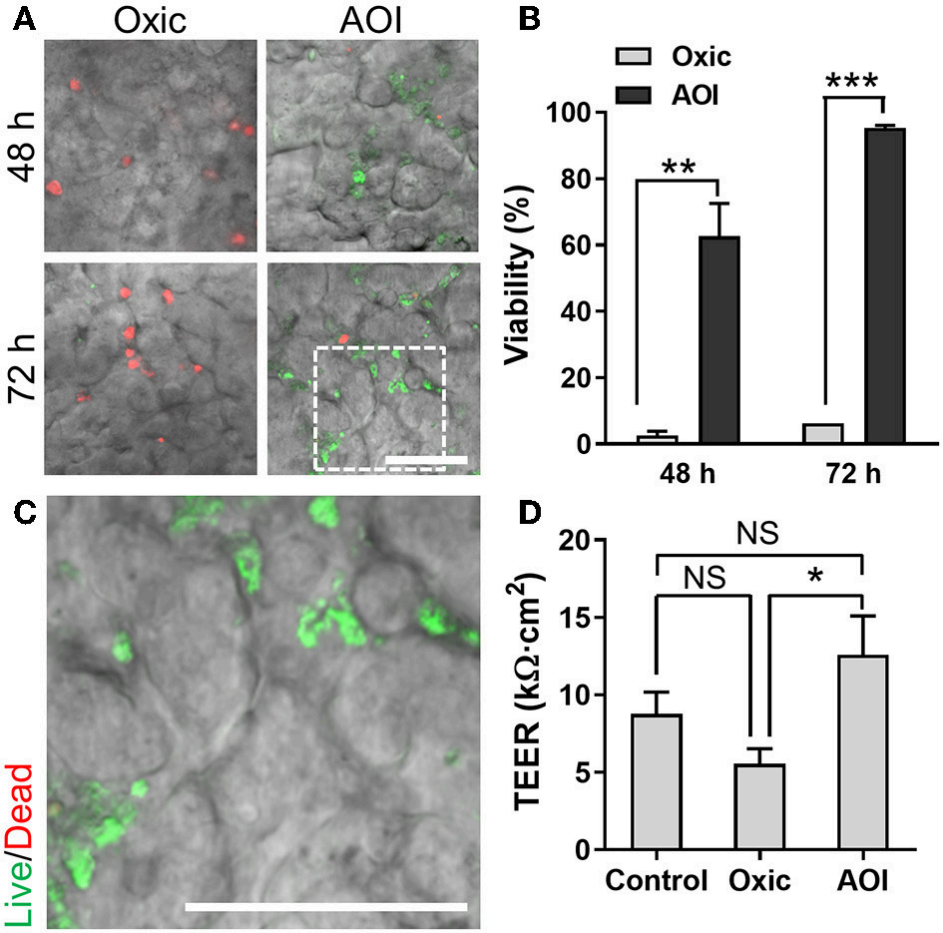
Co-culture of *B. adolescentis* strain with the 3D intestinal epithelial cells in the AOI Chip. **(A)** The viability of *B. adolescentis* cells co-cultured with the epithelium in the AOI Chip visualized by the bacterial Live/Dead assay (live, green; dead, red) in the presence (Oxic) or the absence (AOI) of oxygen in the upper microchannel whereas the lower microchannel is perfused with the oxic medium. Overlaid images are presented by merging a DIC and fluorescence confocal images (red and green channels). For the Live/Dead assay, a mixture of SYTO9 (6 μM) and propidium iodide (30 μM) pre-conditioned in the dark anaerobic glove box was applied into the upper microchannel and assess the bacterial viability assay in a timely manner (< 10 min). **(B)** Quantification of the viability of *B. adolescentis* cells by analyzing the Live/Dead assay results using ImageJ (*N* = 7). **(C)** A zoom-in image cropped from the white dashed square in **(A)** shows the live microcolonies of *B. adolescentis* cells. **(D)** Barrier integrity of the intestinal epithelium grown in the modified gut-on-a-chip at various culture conditions. Control, epithelial monoculture in the AOI Chip; Oxic, co-culture with *B. adolescentis* strain in the oxic chip; AOI, co-culture with *B. adolescentis* cells in the AOI Chip. TEER measurement was performed at 72 h of the co-culture (*N* = 4). NS, not significant. ^*^p < 0.05, ^**^p < 0.001, ^***^p < 0.0001. Bars, 50 μm.

We performed the co-culture of *B. adolescentis* and *E. hallii*, respectively, in the AOI Chip lined by the villous epithelium and conditioned with the anoxic (lumen) and oxic (capillary) culture medium as optimized in Figure 4. After we seeded the *B. adolescentis* cells into the pre-conditioned anoxic upper microchannel, the co-culture setup underwent constant fluid shear stress (0.003 dyne/cm^2^) and mechanical deformations (10%, 0.15 Hz) to maintain the steady-state of nutrient as well as oxygen level in the AOI Chip. After 48 and 72 h of the co-culture, we observed that the majority of *B. adolescentis* cells were dead when they are co-cultured in the oxic medium (Figure 5A, “Oxic”). On the contrary, *B. adolescentis* cells are progressively colonized and expanded the number of a live population in the AOI Chip (Figure 5A, “AOI”), which showed a significant difference in microbial viability compared to the “Oxic” control (Figure 5B). A high-power magnification image reveals that the viable population of *B. adolescentis* cells is found at the intervillus crevice (Figure 5C and Supplementary Figure 4A). We also evaluated the epithelial barrier function by measuring TEER, where the epithelial barrier in the AOI Chip did not show any significant difference from the Control (i.e., the AOI Chip without co-culture with *B. adolescentis* cells). However, the AOI Chip culture showed a significant difference in TEER values from the Oxic control (Figure 5D).

**Figure 6:**
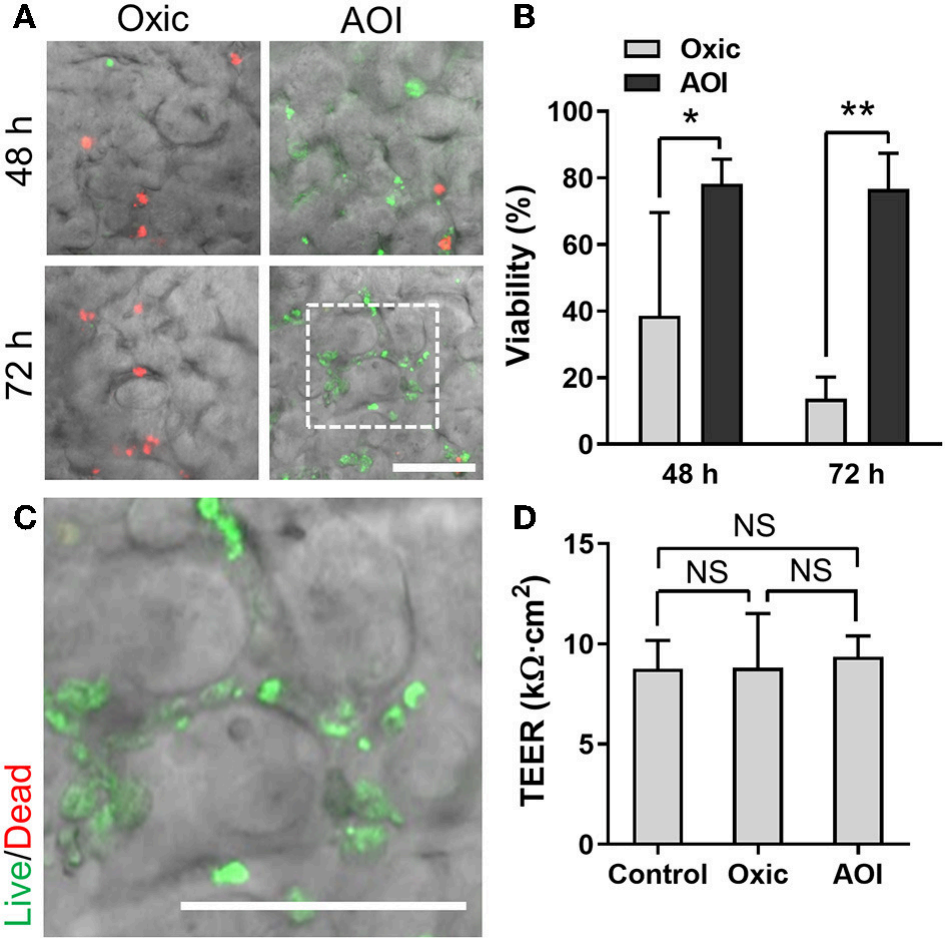
Co-culture of *E. hallii* with the 3D intestinal epithelial cells in the AOI Chip. **(A)** The viability of *E. hallii* cells co-cultured with the epithelium in the AOI Chip visualized by the bacterial Live/Dead assay (live, green; dead, red) in the presence (Oxic) or the absence (AOI) of oxygen in the upper microchannel whereas the lower microchannel was perfused with the oxic medium. Overlaid images are presented by merging a DIC and fluorescence confocal images (red and green channels). For the Live/Dead assay, a mixture of SYTO9 (6 μM) and propidium iodide (30 μM) pre-conditioned in the dark anaerobic glove box was applied into the upper microchannel and assess the bacterial viability assay promptly (< 10 min). **(B)** Quantification of the viability of *E. hallii* cells by analyzing the Live/Dead assay results using ImageJ (*N* = 5). **(C)** A zoom-in image cropped from the white dashed square in **(A)** shows the live microcolonies of *E. hallii* cells. **(D)** Barrier integrity of the intestinal epithelium grown in the modified gut-on-a-chip at various culture conditions. Control, epithelial monoculture in the AOI Chip; Oxic, co-culture with *E. hallii* strain in the oxic chip; AOI, co-culture with *E. hallii* cells in the AOI Chip. TEER measurement was performed at 72 h of the co-culture (*N* = 4). NS, not significant. ^*^p < 0.05, ^**^p < 0.001. Bars, 50 μm.

**Figure 7:**
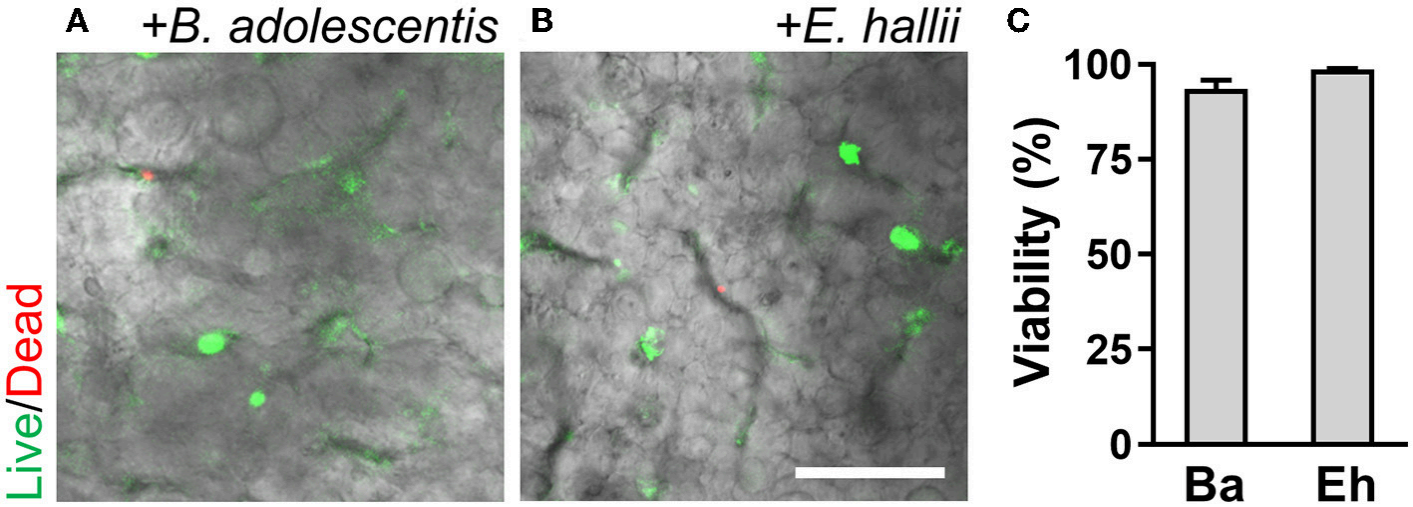
A longitudinal co-culture of the obligate bacteria with 3D intestinal epithelium in the AOI Chip. Bacterial colonies of *B. adolescentis* **(A)** and *E. hallii* **(B)** grown in the intestinal epithelium are highlighted with the fluorescence signals at day 7 of the co-culture. Overlaid images are presented by merging a DIC and fluorescence confocal images (red and green channels). **(C)** Quantification of the viability of bacterial cells by analyzing the Live/Dead assay results using ImageJ (*N* = 4). Ba, *B. adolescentis*; Eh, *E. hallii*. Bar, 50 μm.

To validate the functionality of the AOI Chip, we repeated the same experimental strategy with another commensal gut microbiome, *E. hallii*. This strain is also an obligate anaerobe and the anoxic culture is always required for the colonization (Flint et al., 2007). We found that *E. hallii* cells did not maintain their viability in the microfluidic device under the oxic condition (Figure 6A, “Oxic”), whereas they decently grow and maintained the viable microcolonies in the AOI Chip (Figure 6A, “AOI”). The quantification of cellular viability at 48 and 72 h of the co-culture showed strong evidence that the AOI Chip is necessary and sufficient to maintain the viable population of the obligate anaerobic commensal gut microbiome in the presence of the host epithelium with a statistical significance (Figure 6B). The crevicular growth of the viable *E. hallii* cells over 7 days also revealed the stable coexistence of the host cells and the gut microbiome in the AOI Chip (Figure 6C and Supplementary Figure 4B). We also confirmed that there is no significant difference in the TEER value between groups, indicating that the colonization of the commensal *E. hallii* cells does not compromise the epithelial barrier function (Figure 6D). Finally, we demonstrated that both strains were highly viable more than a week of the co-culture (Figure 7), suggesting that the AOI Chip provides a compelling transepithelial anoxic gradient that allows a robust maintenance of the anaerobic commensal microbial population in the presence of aerobic host epithelium, by which the longitudinal study of the host-microbiome crosstalk can be performed *in vitro*.

## Discussion and conclusion

In this study, we demonstrated an *in vitro* anoxic-oxic interface (AOI) to co-culture the obligate anaerobic gut microbiome with human intestinal epithelial cells in the modified human gut-on-a-chip microphysiological system and analyzed the oxygen gradient generated in the AOI chip by leveraging the computational simulation and experimental validation. The salient finding in this study is the experimental verification of an anaerobic-aerobic interface *in vitro* and the validation of its physiological functionality to support the robust and stable co-culture of obligate anaerobic bacteria with the aerobic host epithelium. We proved that this metabolically challenging co-culture was enabled simply by flowing the preconditioned culture medium without any complicated equipment or facility.

In the colonic microenvironment, anaerobic commensal microbiome colonizes the lumen while oxygen steadily diffuses from capillary vessels toward the lumen (Zheng et al., 2015). Thus, the recreation of this *in vivo* oxygen gradient is necessary to recapitulate the structural (e.g., a lumen-capillary interface) and the functional (e.g., a host-gut microbiome co-culture) configuration in the experimental human intestine model. Reconstituting the intestinal AOI is particularly crucial because stable maintenance of this oxygen gradient is linked to various physiological functions. For example, the homeostatic balance of the oxygen gradient and associated reactive oxygen species (ROS) may play an essential role in regulating the onset of digestive diseases (Espey, 2013). Additionally, the maintenance of an AOI can directly contribute to the stability of colonic host-microbiome crosstalk associated with the production of SCFA by the anaerobic commensal gut microbiome. It has been appreciated that the microbial SCFAs prodigiously orchestrate the metabolic homeostasis of the intestinal epithelium, intestinal barrier function, and regulation of immune functions (Smith et al., 2013; Kelly et al., 2015).

Current experimental models have attempted to recreate this microenvironment, but the metabolically-mismatched co-culture has been challenging to maintain *in vitro*. Conventional static cell culture models are extremely limited in generating an AOI because the culture setup must be incubated in a fixed atmospheric condition (e.g., in an anaerobic glove box or a CO_2_ incubator), which cannot produce and maintain the AOI. For example, a Transwell culture system may temporarily create an oxygen gradient by introducing anoxic and oxic medium to the apical and basolateral side, respectively, but the culture condition in a 5% CO_2_ incubator will rapidly jeopardize the AOI. Furthermore, incubation of the experimental setup in an anaerobic glove box is not appropriate because the introduction of anoxic culture medium substantially compromises cell viability (von Martels et al., 2017). Notably, static cell cultures are limited in demonstrating stable host-microbiome co-culture for an extended period (Kim et al., 2012; Park et al., 2017). Recent microfluidic cell culture models have demonstrated promising results regarding AOI formation *in vitro* (Marzorati et al., 2014; Shah et al., 2016). Introduction of the anoxic and oxic medium into the microbial and epithelial cell microchannels, respectively, established an AOI in both models. However, these models failed to produce a direct contact of the anaerobic microbiome and host epithelium, demonstrated a very short co-culture period (<24 h), and lacked the mechanical deformations that are critical to yield a physiologically accurate intestinal epithelium and sustain stable co-culture (Kim et al., 2016b). The physically segregated compartments in these models also hinder the study of metabolic or pathophysiological crosstalk between the mucosal gut microbiome and the epithelium.

To improve these challenges, we first leveraged the computational simulation to understand the resultant oxygen gradient in a given geometry of the AOI Chip as a function of the linear flow rate and the initial oxygen concentration in the inlet microchannel. We found that a higher linear flow rate may contribute to forming an anoxic or a hypoxic condition in the upper microchannel, suggesting that microfluidic manipulation of the luminal microenvironment may recreate the AOI in our device setup. However, this approach is limited because the flow rate regime in the simulation as well as in the device should accurately mimic the physiological fluidic microenvironment in the colon. The physiological range of the linear flow rate in the ascending colon is approximately 5–30 μm/s (Cremer et al., 2017), and the applied linear flow rate in our experimental model was 27.8 μm/s (equivalent to the volumetric flow rate at 50 μL/h in the microchannel with the height at 500 μm), proving an excellent physiological relevance. Thus, the simple increase of the flow rate in a microfluidic device may not reflect the physiological conditions of the colon. Furthermore, in the absence of an epithelium, the generated AOI was only observed upstream of the microchannels even at a volumetric flow rate 200 μL/h, suggesting that the manipulation of the flow rate alone will not recreate a stable AOI across the entire microchannel. Interestingly, the presence of an epithelial cell layer remarkably changed the oxygen gradient in both the upper and lower microchannels even at a flow rate of 50 μL/h. This observation is mainly attributed to the consumption of oxygen by the epithelial cell layer compared to the simulation result in cell-free conditions. The simulation result also revealed that a steady-state AOI is rapidly established at the upstream edge of the AOI Chip and the mucosal hypoxic condition can be stably maintained across the entire channel in the presence of an epithelial cell layer. Notably, our experimental measurement of oxygen tension showed that the oxygen gradient generated vertically revealed a very sharp change across the epithelial barrier whereas we observed only a gradual change in the computational simulation. This result strongly supports that the biological (e.g., epithelial tight junction), structural (e.g., tall villous height), or physiological factors (e.g., a mucus layer, cytodifferentiation) substantially contribute to the formation of transepithelial AOI in the colon. However, the presence of an epithelial layer alone was also not sufficient to create the AOI in the experimental setup, suggesting that microengineered manipulations, including the flow of anoxic medium and the presence of epithelial cell barrier, are necessary and sufficient for building a stable AOI on-chip. Finally, we did not consider the impact of cyclic strains in the computational simulation. However, we routinely applied the cyclic mechanical deformations in the chip studies, where we did not find any evidence that the cyclic mechanical strain jeopardizes the formation of the anoxic-oxic gradient in the AOI Chip.

To experimentally verify the oxygen profile demonstrated in the computational simulation, we used a commercially available resorufin-based oxygen indicating reagent (Amplex Red) to detect the level of dissolved oxygen in the culture medium in an AOI Chip. As a catalyst, we used platinum-based nanoparticles (Pt-DENs) that performs a peroxidase-like activity. Peroxidases have been found to catalyze the oxidation of various compounds including Amplex Red in the presence of molecular oxygen although the catalyzed oxidation is more dramatic with the addition of hydrogen peroxide (Klapper and Hackett, 1963; Wang et al., 2017). The Pt-DENs have been proved to show a peroxidase-like catalytic activity (Ju and Kim, 2015; Lim et al., 2016), where the nanoscale size of this molecule enabled us to readily mix with the culture medium and introduce into the microchannel. More importantly, since the Pt-DENs are considered to be inert with epithelium without any cytotoxicity, we tested it in our experiments to estimate the *in situ* oxygen level with the Amplex Red. By performing an XZ-vertical real-time microfluorimetric scanning via confocal microscopy, we quantitatively evaluated the concentration of oxygen in the AOI Chip in a spatiotemporal manner. It is noted that we established the steady-state condition by flowing the oxic and anoxic medium independently into the lower and upper microchannels, respectively. Thus, our experimental setup also fully reflects the possible oxygen flux from the PDMS layer into the culture medium *in situ*. Importantly, we did not try to collect the effluent from the chip to measure the oxygen level to avoid inaccurate assessment of the oxygen profile inside the AOI Chip.

We modified the design of the upper (luminal) compartment with 500 μm in height for multiple reasons. First, by increasing the height of the upper microchannel, the intestinal epithelium was allowed to grow up to 300–400 μm (unpublished data). Second, as the cells grow higher, the void volume in the upper microchannel is rapidly reduced, causing a substantial increase in fluid shear stress that is applied to the apical brush border of the epithelium. This high shear stress may cause unintended loss of microbial cells that are adherent on the apical brush border of an epithelial cell layer and also can cause undesired epithelial damage. The original gut-on-a-chip microdevice with an upper microchannel height of 150 μm was designed to emulate 0.02 dyne/cm^2^ of fluid shear stress when the cells form a monolayer (Kim et al., 2012). To compensate for the effects of the high shear stress after the villi formation, we increased the height of the upper microchannel up to 500 μm, which can decrease the shear stress approximately 10 folds. Finally, the increased height of the upper microchannel allowed determining an appropriate volumetric flow rate that is efficiently recreating the physiological transepithelial anoxic gradient. Through the computational simulation, we found that 50 μL/h of volumetric flow rate (equivalent to 27.8 μm/s of linear flow rate in the microchannel at 500 μm in height) was sufficient to form the anoxic gradient in the presence of the epithelial cells. Furthermore, a higher volumetric flow rate generates a sharper anoxic gradient in the AOI chip. As described before, our current experimental setup with 27.8 μm/s of the linear flow rate also well fits within the range of the physiological condition. On the contrary, the upper microchannel with the 150 μm height in the original gut-on-a-chip results in much higher linear flow rate (92.67 μm/s at 50 μL/h) to form the similar profile of the anoxic gradient, which significantly exceeds the physiological linear flow rate.

The live/dead staining of the intestinal epithelium in the AOI Chip strongly suggested that the influx of dissolved oxygen from the lower microchannel is necessary to maintain epithelial cell viability. This biomimetic oxygen manipulation is analogous to the anatomical configuration of the *in vivo* colonic mucosal microenvironments, in which oxygen is perpetually supplied by the underlying capillary vessels (Zheng et al., 2015). Furthermore, no significant difference of MUC2 and ZO-1 expression and localization was found in the villi grown in the AOI Chip, compared to the oxic control, indicating that epithelial barrier functions are well maintained in this transepithelial anoxic gradient. Additionally, epithelial height (*p* = 0.0562; *N* = 3) and the total number of cells (*p* = 0.5019; *N* = 2) are not significantly different between groups in oxic and AOI. In this microengineered colonic microenvironment, we successfully demonstrated the co-culture of the obligate anaerobic gut microbiome with 3D intestinal epithelial cells for up to a week without any loss of epithelial barrier function. Notably, *B. adolescentis* and *E. hallii* strains are known to be extremely sensitive to oxic conditions, and their sustained viability for up to a week-long co-culture demonstrates the efficacy of our AOI chip (Shimamura et al., 1992; Flint et al., 2007). High-resolution confocal imaging analyses also confirmed that the viable population of either *B. adolescentis* or *E. hallii* was colonized between the 3D microarchitecture of epithelial cells. It is noted that the Live/Dead bacterial cell viability assay assessed in a controlled temporal incubation (<10 min) of the assay solution could demonstrate the stochastic spatial colonization of each strain, without any non-specific staining of the epithelium.

In the human gastrointestinal tract, it is believed that the hypoxic gradient is created due to the initial culture of the intestinal mucosa by oxygen-consuming aerobic bacteria, thus allowing subsequent colonization by obligate anaerobes (Espey, 2013). This phenomenon occurs because the oxygen-tolerant microorganisms reduce oxygen tension and oxygen-reduction potential to levels that support the growth of obligate anaerobic bacteria (Savage, 1978). In our study, instead of copying and pasting the whole complexity of intestinal development, we engineered our device to replicate the anoxic gradient in the gut by simplifying the intestinal complexity in a physiologically relevant epithelial tissue interface. We successfully demonstrated the crucial microenvironmental components that are necessary to recreate the AOI in the living human gut such as 3D epithelial microarchitecture, tight junction barrier, and differentiation of MUC2-positive epithelium that contributes to the mucus production. This physiological gut microenvironment enables the formation of an AOI in the microfluidic device after perfusing anoxic and oxic culture media into the upper (luminal) and the lower (abluminal) microchannels, respectively. Although we omitted the colonization of aerobic microbial species, we verified that the microengineered AOI on-chip is necessary and sufficient to manipulate the oxygen gradient accurately. A transepithelial anoxic gradient established with a spatial resolution of ~500 μm around the villous epithelial layer enabled a stable co-culture of obligate anaerobic human gut microbiomes such as *B. adolescentis* and *E. hallii*. By applying the central concept of reverse microengineering, we minimized the biological complexity while still demonstrating the critical elements of a symbiotic ecosystem in the human gut. We believe that our current study shows how to build and validate this physiologically functional circuit to emulate biological complexity.

We selected *B. adolescentis* and *E. hallii* as the representative anaerobic commensal gut microbiome because of their unique contributions to human colon homeostasis. *B. adolescentis* strain predominantly degrades the prebiotic fibers (e.g., inulin or fructooligosaccharides) and produces acetate during the process of fermentation in the colon (Macfarlane and Macfarlane, 2003). *E. hallii* strain utilizes this acetate and produces the SCFA butyrate (Engels et al., 2016). To quantify the potential probiotic effect of these co-cultured bacteria on the viability and the functionality of the epithelial cells, we measured TEER values and determined the epithelial barrier function. Interestingly, the epithelial barrier integrity was significantly increased in the co-culture of *B. adolescentis* in the AOI condition compared to its control culture in the oxic medium (Figure 5D), whereas co-culture with *E. hallii* did not cause any significant difference in the barrier integrity (Figure 6D). This observation is possibly due to the production of SCFA, such as acetate, by *B. adolescentis*, whereas *E. hallii* requires precursor molecules such as acetate to produce other SCFA such as butyrate. Thus, a lack of intermediate metabolic compounds may limit the functionality of the *E. hallii* strain in this experimental format. A future study investigating the co-culture of both species in the AOI Chip may demonstrate the syntrophic cross-feeding between these two strains and exhibit the unique microbial crosstalk that occurs in the gut.

In conclusion, we experimentally and computationally demonstrated that the transepithelial anoxic gradient in the AOI Chip is necessary and sufficient to co-culture the metabolically mismatched obligate anaerobic gut microbiome with the aerobic host epithelial cells. We showed that the oxygen gradient could be successfully recreated and sustained without needing a complicated, resource-intensive apparatus to maintain the anoxic conditions necessary for creating the AOI. Additionally, all experiments were performed in conventional aerobic lab equipment (e.g., a humidified CO_2_ incubator). Thus, our experimental setup does not require the anaerobic glove box to perform AOI-related experiments. The successful co-culture of obligate gut anaerobes in the AOI Chip can potentially lead to studies involving the host-microbiome crosstalk germane to the homeostasis of gastrointestinal functions, regulation of tissue-specific resident immune cells, or the pathophysiology of the intestinal disease. Furthermore, testing the chyme, or indigestible carbohydrates (i.e., prebiotics) with the gut microbiome in our AOI Chip will more closely mimic the chemical microenvironment of the human colon, which can be potential future directions of our study.

## Author Contributions

WS, AW, MM, CF, NT, D-WL, HK, and HJK designed the study. WS, AW, MM, CF, NT, and HJK performed experiments and analyzed data. YJ and JK provided an experimental resource. WS, AW, MM, CF, NT, and HJK wrote and revised the manuscript.

### Conflict of Interest Statement

The authors declare that the research was conducted in the absence of any commercial or financial relationships that could be construed as a potential conflict of interest.
