# Supplementary material for: A Robust Longitudinal Co-culture of Obligate Anaerobic Gut Microbiome With Human Intestinal Epithelium in an Anoxic-Oxic Interface-on-a-Chip
Source: Front Bioeng Biotechnol. 2019 Feb 7;7:13. doi: 10.3389/fbioe.2019.00013 (PMC6374617; doi:10.3389/fbioe.2019.00013)
Supplement: Supplementary file 1 [file Data_Sheet_1.pdf]

*Supplementary Material*

# A Robust Longitudinal Co-culture of Obligate Anaerobic Gut Microbiome with Human Intestinal Epithelium in an Anoxic-Oxic Interface-on-a-Chip

Woojung Shin[1,†], Alexander Wu[1,†], Miles Massidda[1,†], Charlie Foster[1], Newin Thomas[1], Dong-Woo Lee[2], Hong Koh[3], Youngwon Ju[4], Joohoon Kim[4,5], and Hyun Jung Kim[1,6,*]

[1]Department of Biomedical Engineering, The University of Texas at Austin, Austin, USA
[2]Department of Biotechnology, College of Life Science and Technology, Yonsei University, Seoul, Republic of Korea
[3]Department of Pediatrics, Yonsei University College of Medicine, Severance Fecal Microbiota Transplantation Center, Seoul, Republic of Korea
[4]Department of Chemistry, Research Institute for Basic Sciences, Kyung Hee University, Seoul, Republic of Korea
[5]KHU-KIST Department of Converging Science and Technology, Kyung Hee University, Seoul, Republic of Korea
[6]Department of Medical Engineering, Yonsei University College of Medicine, Seoul, Republic of Korea

[†]Equally contributed.

***Correspondence to:**
Hyun Jung Kim, PhD
Department of Biomedical Engineering
The University of Texas at Austin
107 W. Dean Keeton St., BME 4.202C
Austin, TX 78712, USA
Phone: 512-471-2165
Fax: 512-232-4299
hyunjung.kim@utexas.edu

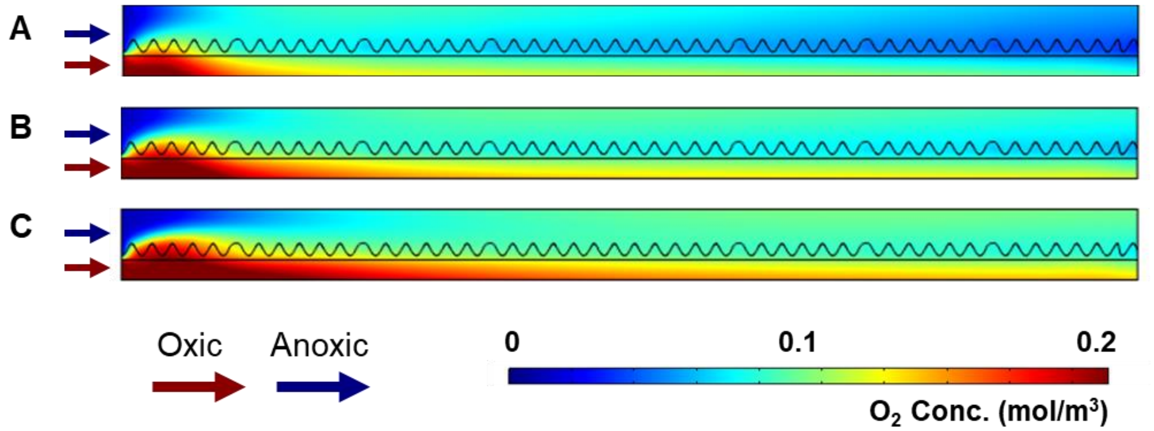


**Supplementary Figure 1.** Generation of oxygen gradient in the AOI Chip by changing the flow rate in the presence of a 3D intestinal epithelial layer. (**A**) A heat map displays the oxygen gradient generated in the two apposed microchannels in the AOI Chip in the presence of a 3D epithelial layer under the flow rate at 50 µL/h. This result is a duplication of the Figure 3B. Heat maps of the computational simulation with the same boundary conditions applied in (**A**) but different flow rates at 100 µL/h (**B**) and 200 µL/h (**C**). A colored scale bar shows the oxygen concentration. Conc., concentration. Arrows indicate the direction of the flow in the microchannel. “Oxic” and “Anoxic” indicate the culture medium preconditioned in a $CO_2$ incubator and an anaerobic glove box, respectively.

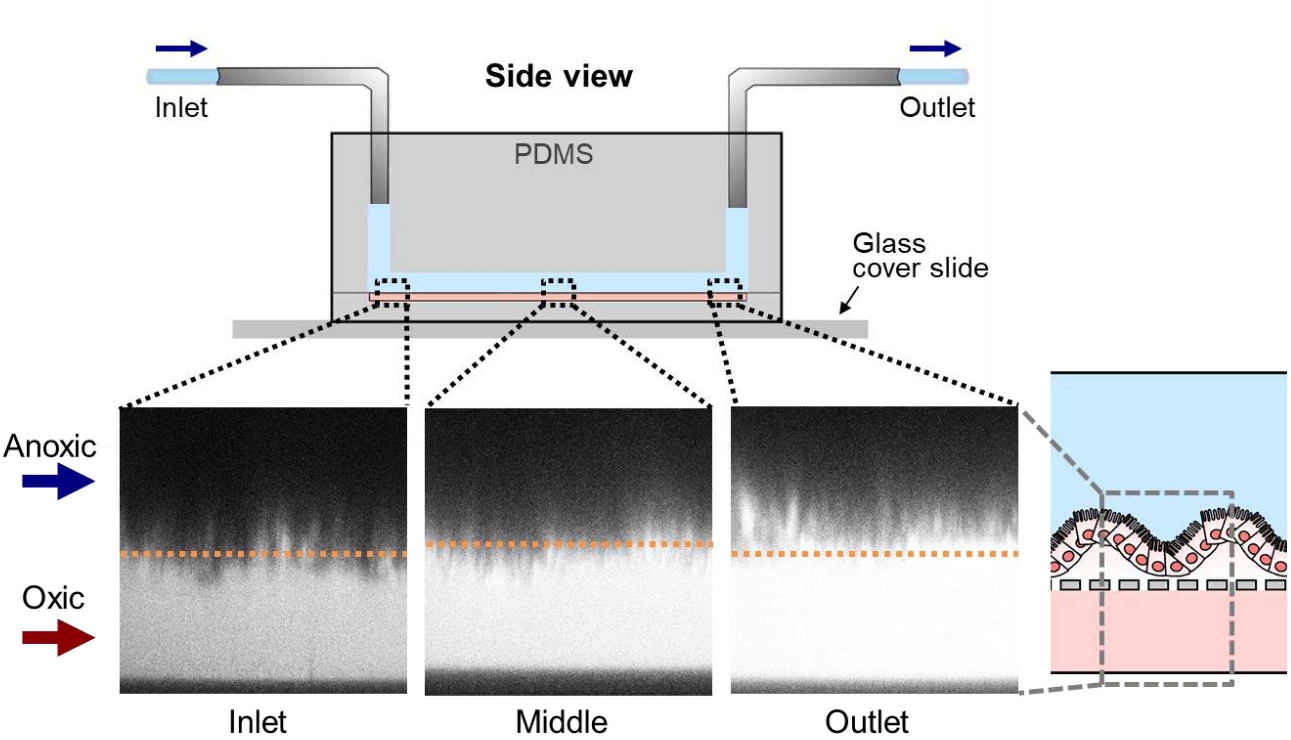


**Supplementary Figure 2.** Visualization of the transepithelial anoxic gradient established in the AOI Chip. Once intestinal epithelium forms 3D villous microarchitecture in the AOI Chip (flow rate at 50 µL/h; mechanical deformation at 10% in cell strain, 0.15 Hz in frequency) for 5 days, the catalyst (Pt-DENs, 3 µM) and fluorescent peroxide detection dye (Amplex Red, 500 µM) were mixed in the anoxic and oxic culture medium and introduced to the upper and lower microchannels, respectively. Volumetric flow rate was constant at 50 µL/h. Fluorescence signal was detected by scanning through the XZ direction after the perfusion for 1 h using a laser-scanning confocal microscopy. The scanned florescence signal was recorded by the acquisition of XZ scanning at each location indicated in the top schematic (black dotted boxes). The yellow dotted lines in the fluorescence images indicate the location of the porous membrane. The acquired vertical position is displayed in the right inset schematic (a grey dashed box).

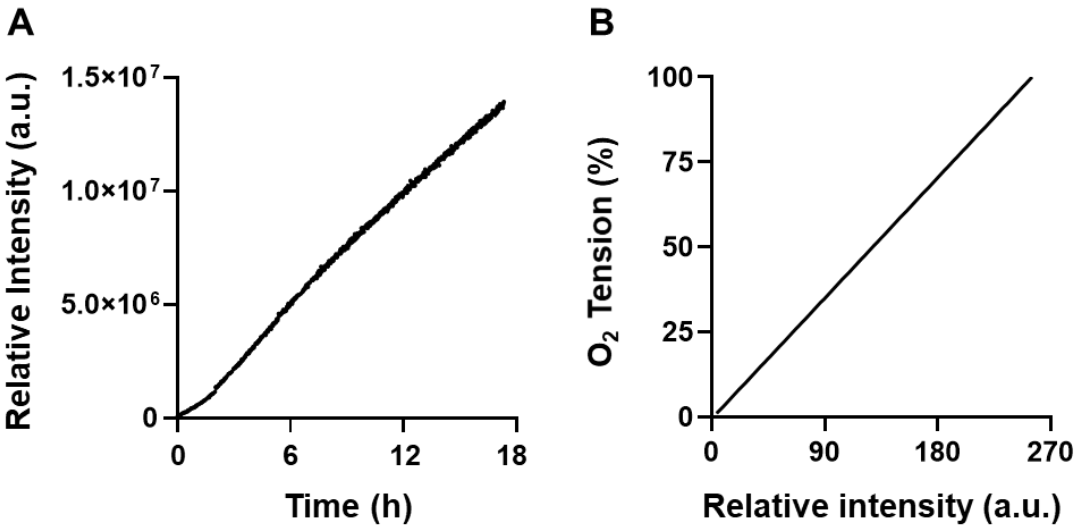


**Supplementary Figure 3.** The calibration of fluorescence intensity to the oxygen tension. (**A**) A kinetic profile of oxygen diffusion in the absolute anoxic cell culture medium in a static 96-well plate. The anaerobic culture medium was prepared by adding $Na_2S$ (6.5 mM), where the Pt-DENs-catalyzed peroxide detection was performed using the Amplex Red reagent at room temperature. Fluorescence intensity was detected in a microplate reader for up to 17.5 h. The anoxic culture medium was oxidized as a function of time. (**B**) A calibration curve plotted with the fluorescence intensity values of the air-saturated (100% $O_2$ tension) and absolute anoxic medium (0% $O_2$ tension) in an AOI Chip at room temperature. Each medium contained Pt-DENs (3 µM) and Amplex Red (500 µM) and imaging analysis was performed instantly after the perfusion of the preconditioned medium into the microchannel using the confocal microscopy. Fluorescence intensity was quantitated by ImageJ. The first-order equation that calibrates the $O_2$ tension and the fluorescence intensity is as follows; "$O_2$ tension" = 0.3944 × "Relative intensity taken by the confocal microscope" - 0.5777. The equation was used to calculate the $O_2$ tension in Figure 3C.

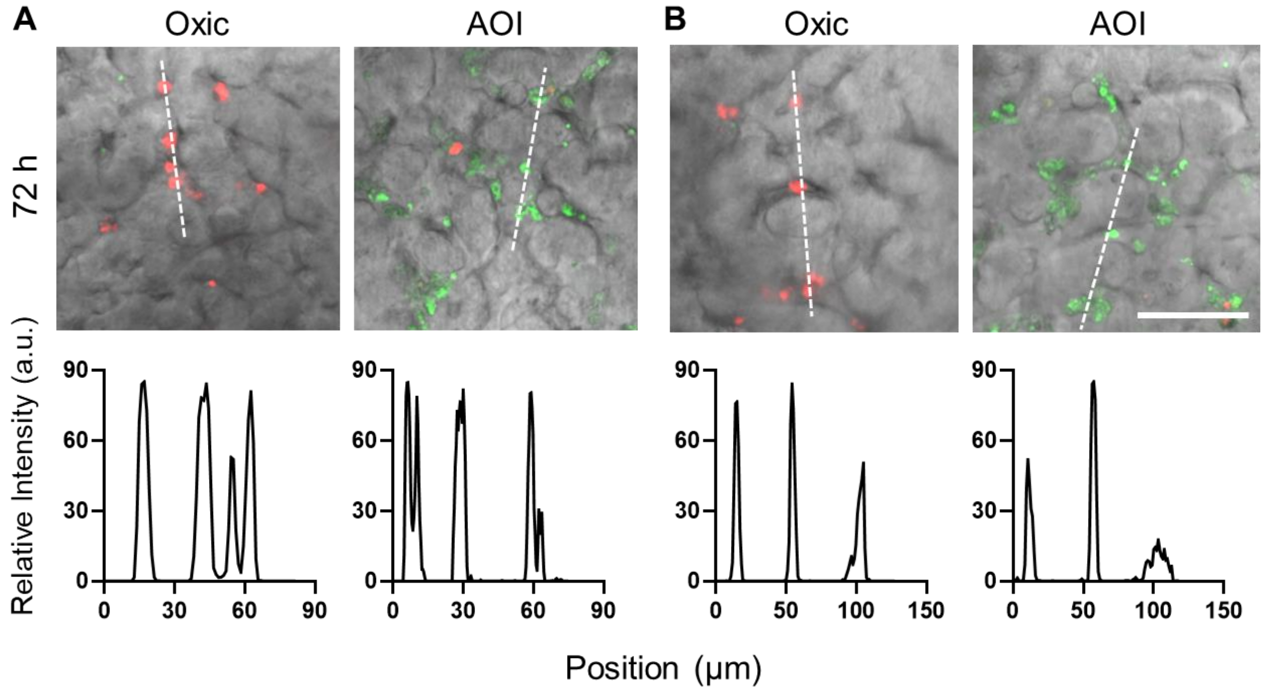


**Supplementary Figure 4.** Intensity profiles of fluorescent images provided in Figures 6 and 7. Arbitrary line scans (white dashed lines) that pass the microcolonies of *B. adolescentis* (**A**) or *E. hallii* cells (**B**) co-cultured with villous epithelium in an AOI Chip (top) and the relative intensity profiles of each overlaid images (bottom). No fluorescence signal was detected in the epithelial region, suggesting that the non-specific staining of the SYTO9/propidium iodide dye was not observed within the detection level. Bar, 50 µm.
